# Assessing the structural connectivity of international trade networks along the “Belt and Road”

**DOI:** 10.1371/journal.pone.0282596

**Published:** 2023-03-08

**Authors:** Wei Chen, Haipeng Zhang, Zhipeng Tang, Zhaoyuan Yu

**Affiliations:** 1 Institute of Geographic Sciences and Natural Resources Research, Key Laboratory of Regional Sustainable Development Modeling, Chinese Academy of Sciences, Beijing, 100101, China; 2 Key Research Institute of Yellow River Civilization and Sustainable Development & Collaborative Innovation Center on Yellow River Civilization Jointly Built By Henan Province and Ministry of Education, Henan University, Kaifeng, 475001, Henan, China; 3 College of Resource and Environment, University of Chinese Academy of Sciences, Beijing, 100049, China; 4 School of Geography Science, Nanjing Normal University, Nanjing, 210023, China; 5 Jiangsu Center for Collaborative Innovation in Geographical Information Resource Development and Application, Nanjing, 210023, China; 6 Key Laboratory of Virtual Geographic Environment, Nanjing Normal University, Ministry of Education, Nanjing, 210023, China; Unviersity of Burgundy, FRANCE

## Abstract

Assessing the trade network connectivity is essential for understanding the trade network structure, optimizing trade development patterns, and improving uneven trade development along the “Belt and Road” (BRI). From the perspective of connectivity, this paper integrates the frontier algorithms in network science and constructs an analytical framework to identify the mesoscale structures, including the community structure, core-periphery structure, and backbone structure embedded in the network, and further explore the structural connectivity of the BRI trade network. The results show that: (1) The BRI trade network represents a trade pattern of “one superpower, many great powers”, with three major trade groups in Southeast Asia, the Middle East, and Northern Central and Eastern Europe in terms of geographical space. China is the super core of the BRI trade network, and the most considerable trade links are all centred in China. (2) Five distinctive trade blocs have formed in the BRI trade network. Nevertheless, the structure of the trade blocs shows significant geographical proximity, indicating that geographical distance still plays a vital role in the international trade system at the regional scale. (3) The BRI trade network demonstrates a significant core-periphery structure, with apparent trade clustering among the core countries within the trade network. Among them, nine countries led by China constitute the core structure, and the peripheral structure is large, reaching forty-four. (4) The trade links with China constitute the backbone structure of the whole trade network in the BRI region. In addition, the trade links related to energy trade and re-export trade are also crucial components of the BRI backbone structure. Methodologically, the analytical framework proposed for assessing the network structural connectivity has great potential to be widely applied to other disciplines and fields.

## 1. Introduction

The “Belt and Road” Initiative (BRI, hereafter) proposed by China in 2013 is committed to building an open cooperation platform for all-around interconnection among countries through infrastructure construction, intergovernmental dialogue and cooperation, economic and trade exchanges, financial integration, and humanistic exchanges. The BRI claims to promote the deep integration of global economic and trade relations and share trade development dividends with a broader range of countries and larger populations [1,2]. Among these objectives, unimpeded trade is a crucial part of promoting the development and cooperation of the BRI [3,4]. To achieve unimpeded trade, trade connectivity needs to be continuously enhanced. High levels of trade connectivity are essential because they lead to fewer barriers to the occurrence and development of trade, a greater and faster flow of trade between countries, and more efficient foreign trade for economic development [5]. The level of trade connectivity determines the effectiveness of trade interactions. Therefore, the goal of the BRI in promoting trade development is to achieve joint development by enhancing the trade connectivity level of the BRI countries [6]. A reasonable assessment of the trade network connectivity of the BRI countries is an important tool for understanding the characteristics of trade interactions between countries, which is necessary for strengthening trade relations, optimizing trade network structure, and promoting regional economic and trade integration and the sustainable development of the BRI.

The relationship between globalization and regionalization of international trade is an important topic in the study of international trade [7,8]. With the continuous advancement of research methods and the wide application of interdisciplinary research tools, scholars have generally used complex network analytical methods to portray the structure of trade networks and trade grouping characteristics among countries since the 1990s [9]. For instance, Serrano et al. used a complex network approach to verify the small-world and scale-free properties of the world trade network [10]. Based on the stem-branch relationship in cyberspace, Snyder et al. identified the core-periphery structural characteristics of inter-country trade networks [11]. Garlaschelli et al. further demonstrated that the core and periphery positions of trade networks are spatially correlated [12] and that the position of a country in a trade network determines its position in the global value chain [13]. By introducing centrality and community coreness indicators, Federica et al. tried to identify the key industries in the world’s import and export trade network [14]. Network topology is also a major concern for trade networks, and Fagiolo et al. have characterized the evolution of world trade networks based on a weighted network perspective [15]. In addition to research on the overall pattern of trade networks, international academic research on specific industries or product types has also been a hot academic issue. Scholars have conducted research on trade networks for particular products such as energy and minerals [16], agricultural products [17], and rare earths [9], revealing the changing status of countries with different leading industries and resource endowments in specific trade networks. Developing related research has laid a solid foundation for enriching and expanding the connotation of international trade network analysis.

With the introduction of the BRI, the emerging BRI trade network has given new impetus to studying international trade networks under the framework of constructing free, open, and inclusive global trade relations [18]. Sui et al., for instance, conducted a comparative study of trade and value-added trade along the BRI based on the network analysis perspective [3]. Similarly, Song et al. used association analysis to compare the BRI trade network and its topological relationship with the global trade network [6]. With the help of the top-level trade network idea, Liu et al. also identified the trade relationship structure and the temporal and spatial evolution characteristics of countries along the B&R based on the backbone trade network [4]. In addition to studies on the overall scope of the BRI, some scholars have conducted in-depth discussions on the trade network between China with countries in specific regions along the BRI route, including Transcaucasia [19], West Asia [20], and Indo-China Peninsula [21]. Moreover, emerging studies focus on trade networks for specific commodities in countries along the B&R, such as food, new energy vehicles, marine energy products, and oil.

At present, research progress has been made on the pattern, structure, and influence mechanism of international trade networks and the trade networks of specific commodity types. As a new international economic cooperation platform, the Belt and Road region has also become a popular research area for international trade network research. However, the following research gaps still exist in the existing studies. 1) Insufficient attention has been paid to the connectivity changes in trade networks led by the BRI, and little literature exists that focuses on the assessment and quantitative measurement of the connectivity of the BRI trade network. More importantly, assessing the connectivity of the BRI trade network is an essential approach for us to comprehensively and scientifically understand the pattern, topology, and structural characteristics of the BRI trade network. 2) Scholars have begun to emphasize the application of network analysis in the study of international trade networks. Still, the update and application of frontier algorithms in network analysis have lagged relatively behind. Nevertheless, with the tremendous progress in network analysis, many insightful and targeted network analytical methods have been proposed, which can be applied as tools for characterizing trade network structures and their connectivity more precisely.

This study contributes to the literature in three respects. First, "the Belt and Road" has attracted great attention as a new international economic cooperation platform. In this paper, we take the Belt and Road region as a case study to enrich the understanding of economic and trade cooperation of the BRI trade network; Second, we identify the mesoscale structures in the BRI trade network, including community structure, core-periphery structure, and backbone structure, which makes up for the shortcomings of current studies that focus on macroscale and microscale trade networks; Third, we integrate various network analysis methods to build an analytical framework for measuring trade network connectivity, which has great potential to be applied to other disciplines.

Therefore, to fill this gap, this paper integrates the frontier algorithms in network science and constructs an analytical framework to comprehensively assess the trade patterns, topological relationships, and structural characteristics of the BRI region from the two dimensions of nodes and edges, finally evaluating the structural connectivity of the BRI trade network. We believe this study could provide a reference for decision-making to improve trade development further and promote the joint development between countries along the “Belt and Road”.

## 2. Methodology and data

### 2.1 Study area

The BRI is committed to building an inclusive and open cooperation platform and is not limited to a specific geographical space. However, for the sake of practical research, the spatial scope of the Belt and Road Initiative still needs to be clarified. With reference to the geographical scope of previous studies on the BRI region[3,4,6], the geographical scope of the study on the BRI trade network refers to the traditional 65 countries along the Belt and Road (that is, the countries along the ancient Silk Road) [1].

### 2.2 Analytical framework

As an open and inclusive international cooperation platform, the BRI has attracted significant attention from governments and academic researchers. Consequently, among the BRI issues, trade cooperation is a priority field [4]. To this end, in this paper, we attempt to integrate the mainstream and advanced network analysis methods related to the mesoscale structure embedded in real-world networks and build an analytical framework to evaluate the structural connectivity of international trade networks among BRI countries and regions.

In the first step, to visualize the spatial network of international trade along the BRI, we constructed a trade network matrix of 65 countries along the BRI and revealed the overall pattern of the Belt and Road trade network from a geographical perspective. Secondly, we extracted the top 3 network of the BRI trade network to capture the most important international trade relations and then adopted the Leiden community detection algorithm to identify the community structures in the BRI trade network. Furthermore, we applied the core-periphery profile algorithm to analyze the core and peripheral structures and identify the most influential and peripheral countries and regions in the BRI trade network. Finally, we employed the backbone extraction algorithm for the weighted network to identify the backbone structure of the BRI trade network. Based on the above analytical framework, this paper aims to comprehensively assess the overall structural connectivity of international trade networks along the Belt and Road.

### 2.3 Data processing

The trade data used in this paper comes from UN Comtrade (United Nations Commodity Trade Statistics Database, https://comtrade.un.org/data/), created by the United Nations Statistics Department (UNSD). The UN Comtrade is considered the world’s largest and most authoritative international commodity trade data-based resource and includes international commodity trade flows for more than 200 countries and regions.

To reveal the structural characteristics of the BRI network, we needed to construct a matrix of trade links between countries across the Belt and Road. However, maintaining the accuracy and consistency of trade data between countries is not always easy due to differences in statistical caliber, calculation methods, and even processing errors worldwide. Countries and regions generally monitor imports more closely than exports [22], so import data is believed to be more accurate than export figures [13,23]. Based on the UN Comtrade Database, we extracted the trade flow data based on import caliber among countries and regions, including the attributes of importing countries, exporting countries, trade volume in commodities, and flow direction, and constructed the trade network of the BRI countries. In addition, some countries still had missing trading statistics that needed to be accounted for. To solve this problem, based on the trade data constructed from the import caliber, we supplemented and improved the trade data of the countries with missing data by introducing export data. Finally, the BRI trade network was constructed by a weighted and undirected matrix containing 65 countries according to the BRI region in 2019.

Since the global COVID-19 pandemic broke out at the end of 2019, the global economy and trade systems have suffered a significant impact, and the world economy is still experiencing a downturn. In light of this fact, we used data from 2019 in this article to study the structural characteristics of the BRI trade network.

### 2.4 Methods

#### 2.4.1 Community detection

In complex networks, a community refers to a subset of a network. The nodes can be grouped into nodes that each community is closely connected to internally with sparser connections between groups, called community structure [24,25]. Modularity is one of the best-known quality measures for community detection [26]. This method maximizes the difference between the actual number of edges in a community and the expected number of such edges. Optimizing modularity is not always easy, and many algorithms have been proposed.

Among these algorithms, the Louvain algorithm [27] is one of the most popular and high-performing techniques for modularity optimization. However, the Louvain algorithm is apt to yield poorly connected or even disconnected communities when run iteratively [28]. To address this problem, the Leiden algorithm was introduced to remedy the drawback of the Louvain algorithm. It takes advantage of the idea of speeding up the local moving of nodes and the idea of moving nodes to random neighbours and then guarantees communities well connected with higher modularity and higher computational efficiency.

Specifically, the Leiden algorithm is run iteratively and converges to a partition in which all subsets of all communities are guaranteed to be locally optimally assigned. Let *G* = (*V*, *E*) be a graph with *n =* |*V*| nodes and *m =* |*E*| edges. A partition *P* = {*C*_1_,…,*C*_*r*_} consists of *r =* |*P*| communities, where each community *C*_*i*_⊆*V* consists of a set of nodes, and a set of S constitute a Community C. The guarantees of the Leiden algorithm partly rely on the randomness in the algorithm. In this definition, *P* is a flat partition of a graph *G* = (*V*, *E*). For a set of nodes *S*⊆*C*∈*P*, any subset of nodes in a community is always connected to the rest of the community with a density of at least *γ*. This implies that

E(S,C−S)≥γ‖S‖∙‖C−S‖ (1)

Uniform *γ*-density implies subpartition γ-density, which in turn implies *γ*-connectivity. The two properties of *γ*-separation and γ-connectivity are guaranteed in each iteration of the Leiden algorithm. Therefore, this paper employs the Leiden community detection algorithm to reveal the trade blocs embedded in the BRI trade network.

#### 2.4.2 Core-periphery profile

The conception of a network as divided into a dense core and a sparse periphery, referred to as a core-periphery structure [29]. Some algorithms were proposed successively, however, most of the proposed algorithms are unable to deal with the weighted networks, and their robustness still needs to be verified. Against this background, Della Rossa et al. recently proposed the algorithm of core-periphery profile [30], disclosing the overall network structures and the peculiar roles of specific nodes.

In most real-world networks, however, the core-periphery structure is not ideal, though the core-periphery structure is evident: a weak (but not null) connectivity exists among the peripheral nodes. This calls for the generalized definition of α-periphery, which denotes the largest subnetwork S with the persistence probability *α*_*S*_≤*α*.

We define the network’s core-periphery profile *α*_*k*_, *k = 1*, *2*, *…*, *n*, using the following algorithm. This is the equation:

$$\alpha_{k}=\underset{h\in N\backslash P_{k-1}}{\min}\frac{\sum_{i,j\in P_{k-1}\cup \{h\}}\pi_{i}m_{ij}}{\sum_{i\in P_{k-1\cup \{h\}}}\pi_{i}}$$

$$=\underset{h\in N\backslash P_{k-1}}{\min}\frac{\sum_{i,j\in P_{k-1}}\pi_{i}m_{ij}+\sum_{i\in P_{k-1}}\left(\pi_{i}m_{ih}+\pi_{h}m_{hi}\right)}{\sum_{i\in P_{k-1}}\pi_{i}+\pi_{h}}$$ (2)

We start with the node *i* that has the weakest connectivity, and generate a sequence of sets *{1} = P1 P2 … Pn = N* by adding, at each step, the node attaining the minimal increase in the persistence probability. Correspondingly, we obtain the core-periphery profile, that is, the sequence 0 = *α*_1_≤*α*_2_≤⋯≤ *α*_*n*_ = 1 of the persistence probabilities of the sets *P*_*k*_.

The above algorithm provides two other important tools of analysis as byproducts: centralization and coreness. We define the centralization C for a core-periphery profile *α*_*k*_ as the complement to 1 of the normalized area, namely:

$$C=1-\frac{2}{n-2}\sum_{k=1}^{n-1}\alpha_{k}$$ (3)

We can therefore quantify such similarity by measuring the area between the *α*_*k*_-curve of a given network and that of the star network and normalizing to assign C = 1 to the star network itself (maximal centralization) and C = 0 to the complete network (no centralization).

#### 2.4.3 Disparity filter

Backbone structure is also a critical hidden mesoscale structure in complex networks. A network’s backbone is a sparse and (un)weighted subgraph that contains only the most ‘important’ or ‘significant’ (weighted) edges [31,32]. Backbone structure can be helpful when the original network is too dense or edge weights are difficult to interpret. Among the proposed methods, the disparity filter algorithm [33], exploits local heterogeneity and local correlations and is able to filter out the backbone of dominant connections in weighted networks with the strong disorder.

To assess the effect of inhomogeneities in the weights at the local level, for each node *i* with k neighbours, one can calculate the function:

$$\omega_{i}(k)=kY_{i}(k)=k\sum_{j}p_{ij}^{2}$$ (4)

where *Y*_*i*_(*k*) characterizes the level of local heterogeneity. Under perfect homogeneity, when all the links share the same amount of the strength of the node, *ω*_*i*_(*k*) equals 1 independently of *k*, while in the case of perfect heterogeneity, when just one of the links carries the whole strength of the node, this function is *ω*_*i*_(*k*) = *k*.

The null model that we use to define anomalous fluctuations. The probability density function for one of these variables taking a particular value x is:

$$\rho (x)dx=(k-1){\left(1-x\right)}^{k-2}dx$$ (5)

which depends on the degree k of the node under consideration.

The disparity filter proceeds by identifying which links should be preserved for each node in the network. The null model allows this discrimination by the calculation for each edge of a given node of the probability *α*_*ij*_ that its normalized weight *p*_*ij*_ is compatible with the null hypothesis. The statistically relevant edges will be those whose weights satisfy the relation

$$\alpha_{ij}=1-(k-1)\int_{0}^{p_{ij}}{\left(1-x\right)}^{k-2}dx<\alpha$$ (6)

Note that this expression depends on the number of connections k of the node to which the link under consideration is attached.

## 3. Results

### 3.1 Trade network pattern of the BRI region

**Fig 1:**
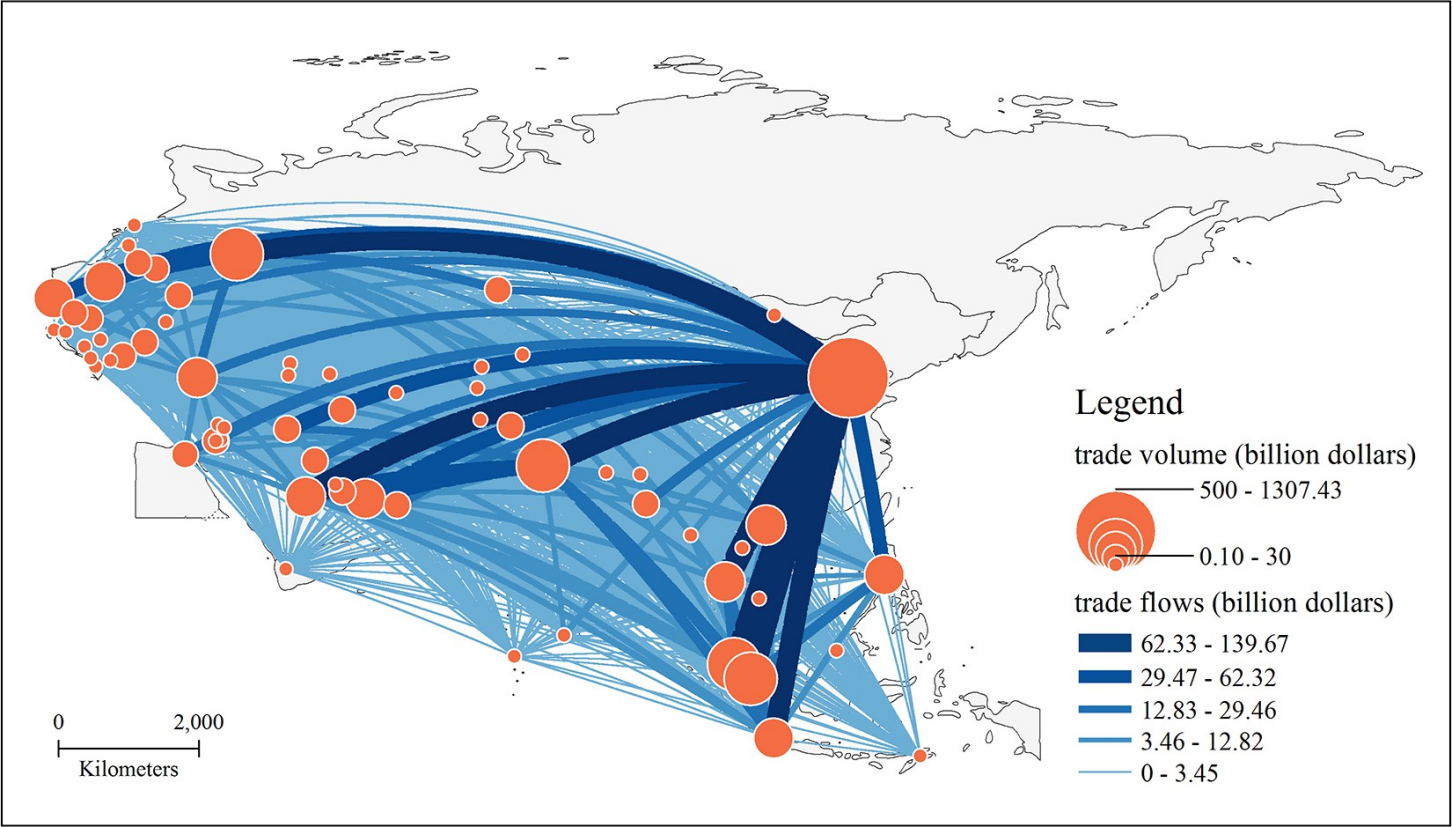
The trade network pattern of the BRI region. Note: The basemap used to construct the map was obtained from the following source: China Map Publishing House Co., LTD. They have been republished under a CC BY license, with permission from the China Map Publishing House Co., original copyright [2021].

In this paper, the BRI countries are taken as the nodes in the trade network, and the total trade volumes of each country to 65 countries are used to indicate the weight of the connected edges in the network, and no limit is set on the scale of bilateral trade flow. In other words, the trade links are established when there are trade flows to build a complete non-directional trade network along the B&R (Fig 1). The study found that after nearly six years of the development of the BRI, a tightly connected trade network has been established among countries along the BRI.

In terms of total trade, the BRI trade network has the following characteristics: 1) The trade network has formed the trade pattern that reflects the concept of “one superpower, many great powers” and the trade share is more concentrated. China is the super core of the trade network. Its total trade with countries along the route reached 1.31 trillion U.S. dollars, accounting for 23.3% of the total trade network of countries along the BRI, China’s trade volume far exceeds that of other countries. India, Russia, Malaysia, and Singapore make up the vital core of the BRI trade network, with each country’s total trade to the countries along the route accounting for more than 5% of the entire trade network. The total trade volume of the top 5 countries accounts for nearly 50% of the total trade volume along the BRI. 2) The trade network presents three high-value trade accumulation areas in geographic space: Southeast Asia, the Middle East, and the northern region of Central and Eastern Europe. Among them, the larger trade nodes in Southeast Asia are Malaysia, Singapore, Vietnam, Thailand, and Indonesia, and the economic development is characterized by significant foreign trade dependence. The UAE, Saudi Arabia, and Turkey are large trade nodes in the Middle East. These countries are rich in oil and other mineral resources and are the main exporters of mineral resources along the BRI. The total trade volume of Poland, the Czech Republic, and Belarus in the northern part of CEE is larger because of the generally positive economic development of these countries in recent years, while the total foreign trade volume continues to climb as the trade potential with China continues to be trapped under the framework of China-CEE cooperation.

The trade flows possess the following characteristics: 1) Trade links between large countries in the trade network are extremely strong, and the connections with the largest flow sizes are centred in China. Large-scale trade flows are common between China and large countries such as Russia, India, Saudi Arabia, and Turkey. China’s trade with Vietnam, Russia, and Malaysia generate more than 100 billion dollars, making these trade links the strongest BRI countries. 2) Extensive trade connections are common among the nodes of the trade network, even if the trade scale between some countries is small. Maldives, Bhutan, and Timor-Leste are the three smallest countries in the BRI trade network and have established stable trade ties with China. The total number of countries with which they have trade relations is nearly one-half of the total number of the BRI countries.

### 3.2 Community structure of the BRI trade network

**Fig 2:**
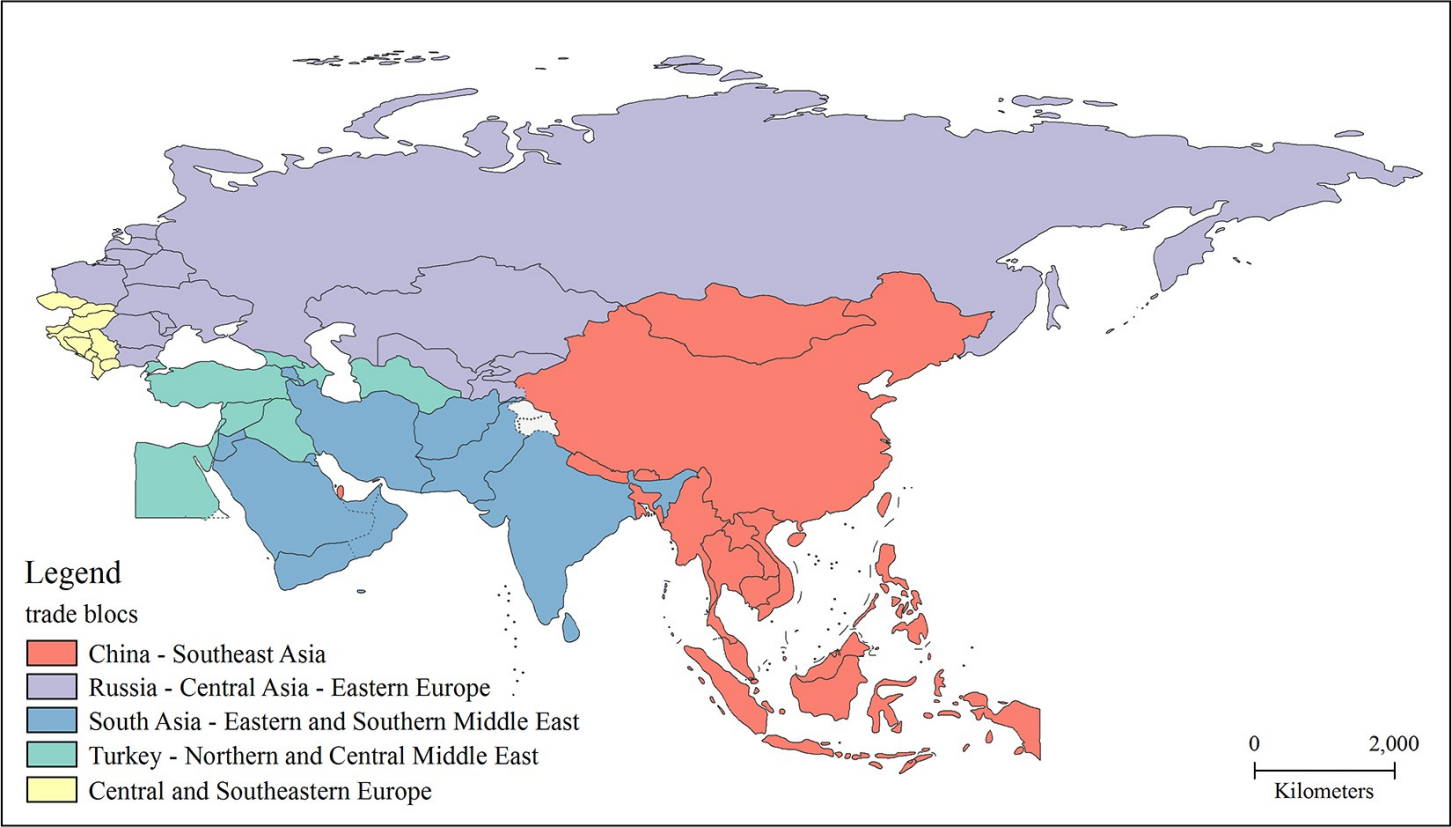
Community structure of the BRI trade network. Note: The basemap used to construct the map was obtained from the following source: China Map Publishing House Co., LTD. They have been republished under a CC BY license, with permission from the China Map Publishing House Co., original copyright [2021].

According to the above analysis, the trade networks vary significantly concerning trade volume between countries, which is not conducive to demonstrating a clear network structure. Therefore, many scholars have adopted the top network approach to extract the main structure from the whole trade network, reducing the obscuration of trade information between large and small countries [4]. Although the top network loses some trade links, it still reflects the backbone structure of the entire network. Therefore, in this paper, we adopt the top network approach, in which each country retains its top trade links in terms of trade volume, and the calculation results of all countries are combined. Finally, the top 3 trade network of the BRI region are extracted, and the Leiden community detection method is then used to figure out the community structure of the BRI trade network. In the context of globalization and regional integration, five distinctive trade groups have formed in the BRI trade network (Fig 2). Furthermore, the community structure shows significant geographical proximity characteristics, indicating that geographical distance still plays a crucial role in the international trade system at the regional scale.

#### (1) China-Southeast Asia Cluster

This community is the largest trade group in the BRI trade network, forming a single core network structure with China as the absolute core. This trade group is made up of 18 countries, including China, Bangladesh, Brunei, Bhutan, Indonesia, Cambodia, Laos, Maldives, Myanmar, Mongolia, Malaysia, Nepal, Philippines, Qatar, Singapore, Thailand, Timor-Leste, and Vietnam. The total trade within the trade group reached 925.71 billion dollars. The countries within this group are mainly ASEAN countries with highly close trade relations with China, are geographically adjacent to China, and have a similar history, culture, customs, and ethnic traits. In addition, the China-ASEAN FTA launched in 2010 has intensified trade interactions and increasingly close trade ties between ASEAN countries and China.

#### (2) Russia-Central Asia-Eastern Europe Cluster

This community is the third-largest trade group in the BRI trade network, and the trade volume reached 75.40 billion dollars. Russia is the core of this monocentric trade group. Other countries include Bulgaria, Belarus, Estonia, Kazakhstan, Kyrgyzstan, Lithuania, Latvia, Moldova, Poland, Romania, Tajikistan, Ukraine, and Uzbekistan, for a total of 14 countries. This trade group is dominated by former Soviet Union countries, which are geographically close to each other and have a shared history, a complementary solid industrial system, close economic and trade relations with Russia, and a compact trade group structure.

#### (3) South Asia-Eastern and Southern Middle East Cluster

The community is the second largest trade group in the BRI trade, with a trading volume of 187.92 billion dollars. The basic formation of this trade group consists of a multi-core trade network with India, UAE, and Saudi Arabia as the primary nodes that also includes Afghanistan, Iran, Armenia, Bahrain, Jordan, Kuwait, Sri Lanka, Oman, Pakistan, and Yemen for a total of 13 countries. These countries are geographically close and rich in energy resources, but their trade products are relatively single. As a result, their trade links are also relatively loose, and the risk of trade structure stability is high.

#### (4) Turkey-Northern and Central Middle East Cluster

This trade group is a monocentric network structure with Turkey as the core node, including Azerbaijan, Egypt, Georgia, Iraq, Israel, Lebanon, Palestine, Syria, and Turkmenistan for ten countries. The total trade volume is just about 36.90 billion dollars. The trade group shows a more significant influence of geopolitical ties on trade relations. Among the three Transcaucasian countries, Azerbaijan and Georgia have closer ties with Turkey, while Armenia, a neighbour of Turkey, has weaker economic and trade relations with Turkey due to its political rivalry with the two countries. Armenia is thus not included in this group.

#### (5) Central and Southeastern Europe Cluster

This trade group includes Albania, Bosnia and Herzegovina, the Czech Republic, Croatia, Hungary, North Macedonia, Montenegro, Serbia, Slovakia, and Slovenia of 10 countries. The trade volume exceeds 70 billion dollars. The cluster does not have a strong trade core to lead and presents a multi-node network structure. The countries within the trade group are generally small in trade volume and have relatively loose trade ties. Still, they have experienced a positive economic situation in recent years, including strengthening trade relations with China that have stimulated their domestic economic growth significantly. Therefore, the trade relations within this group are expected to be further strengthened in the future.

### 3.3 Core-periphery structure of the BRI trade network

**Fig 3:**
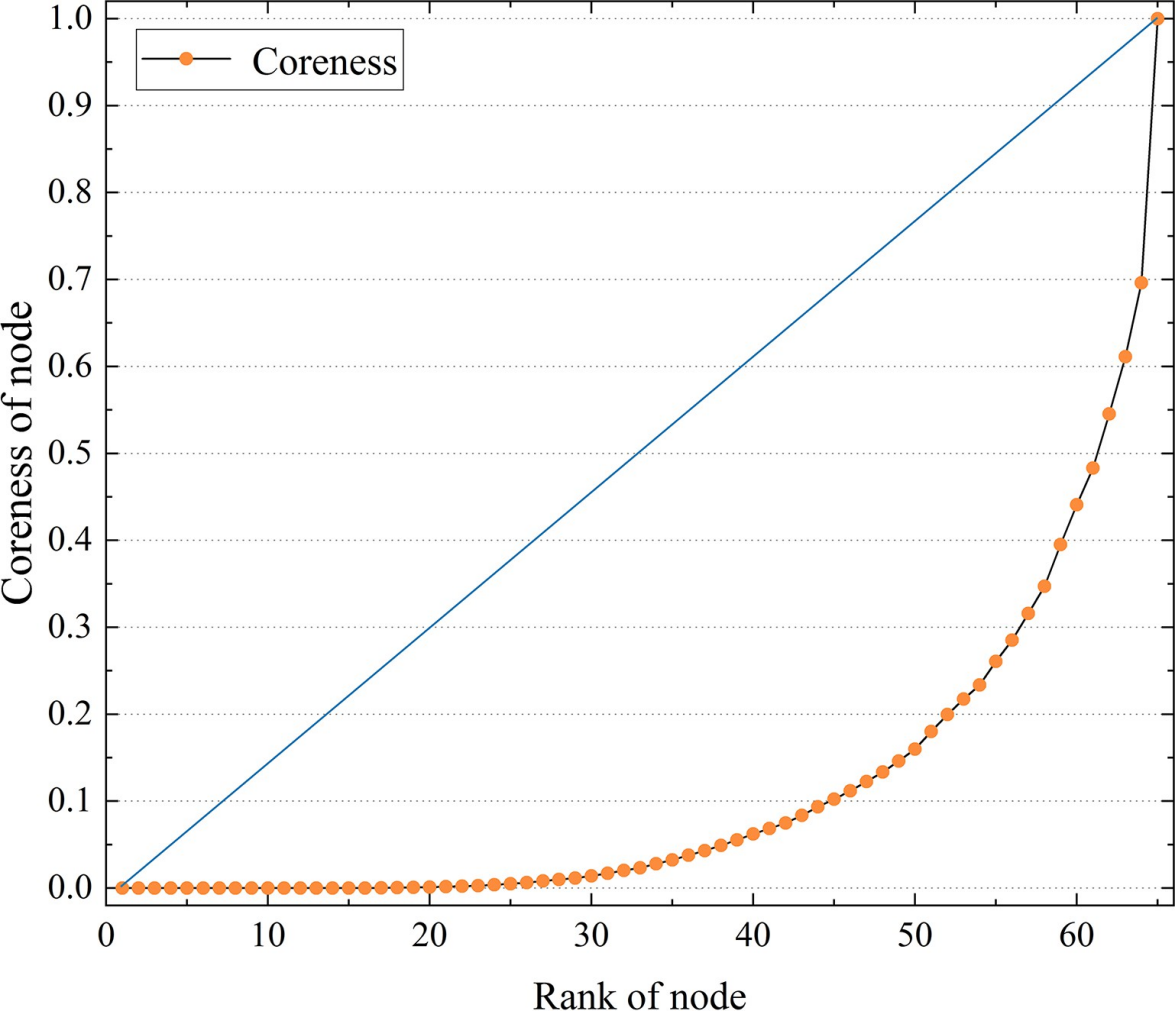
The relationship between coreness and rank of the countries in BRI trade network.

In the core-periphery profile algorithm, the coreness and concentration coefficient are the most important indicators to reveal the core-periphery characteristics. The coreness demonstrates the position or role of individual nodes in the network, and the concentration coefficient measures the polarization effect of the core-periphery structure in the network. The concentration coefficient of the BRI trade network reaches 0.79, indicating a significant core-periphery structure exists in the BRI trade network. The agglomeration effect of the core countries within the trade network is pronounced, with some nodal countries occupying a vital position in the network. Some nodal countries occupy important positions in the network. Fig 3 shows the coreness comparison of the core-periphery structure of the BRI countries. The horizontal axis shows the node order of each country according to the coreness, and the vertical axis shows its corresponding coreness. As can be seen from the curves in the figure, the core degree of nodes gradually diverges when the bit order of nodes exceeds nearly half of the total number of nodes. The relationship between the coreness and rank of the BRI countries shows a “J” type curve (i.e., the coreness of nodes with high bit order is exceptionally high and the number is small; the core degree of nodes with low bit order is generally low, and the number is large).

**Fig 4:**
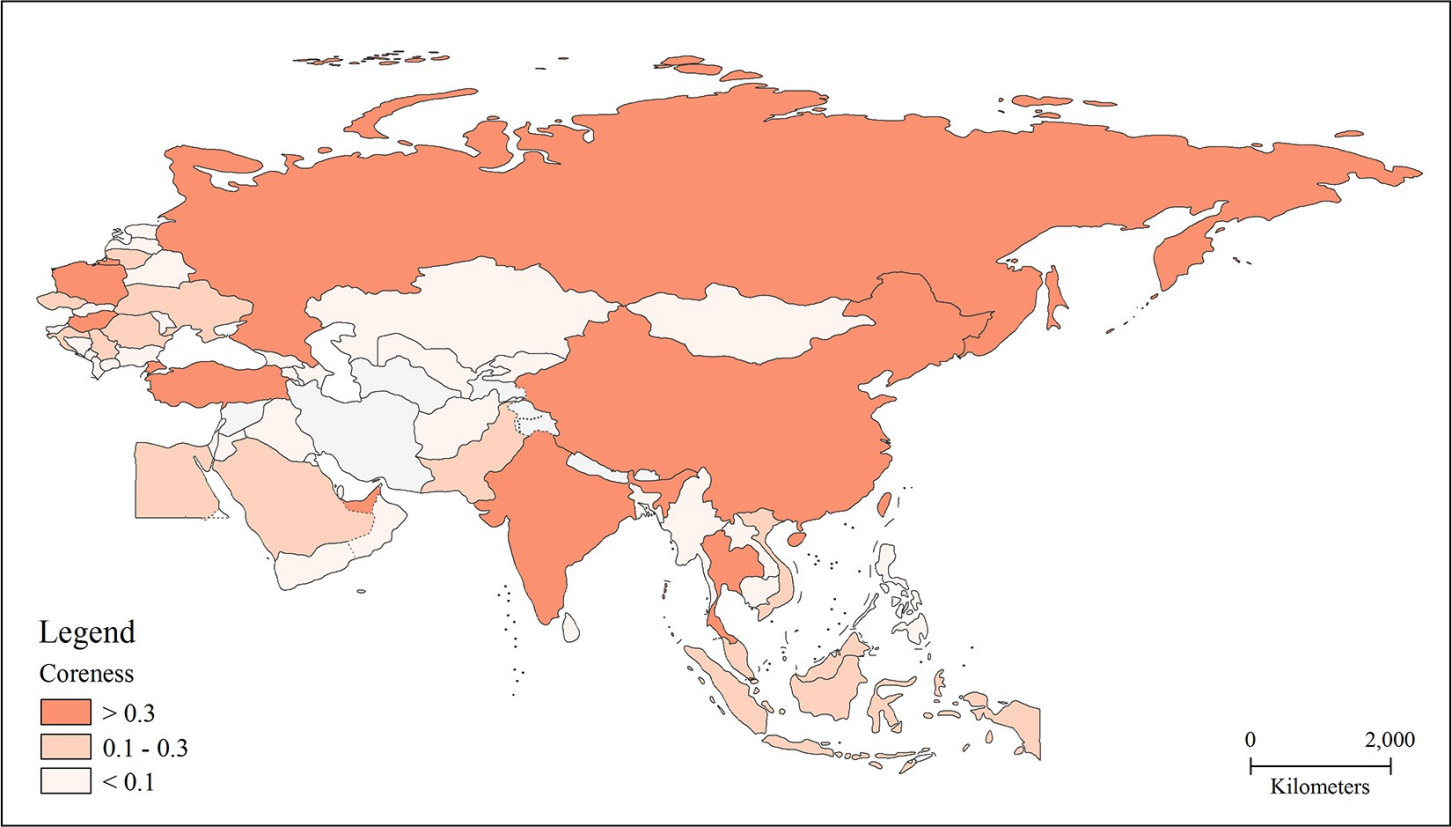
Core-periphery structure of the BRI trade network. Note: The basemap used to construct the map was obtained from the following source: China Map Publishing House Co., LTD. They have been republished under a CC BY license, with permission from the China Map Publishing House Co., original copyright [2021].

To further analyze the core-periphery status of the BRI trade network, we classify the countries with coreness greater than 0.3 as the core structure, the countries with coreness between 0.1 and 0.3 as the semi-peripheral structure, and the countries with coreness less than 0.1 as the peripheral structure (Fig 4). Nine countries are located in the absolute core structure, including China, India, Russia, Singapore, Poland, Thailand, Turkey, the United Arab Emirates, and Hungary. Among them, the countries with a coreness greater than 0.5 are China, India, Russia, and Singapore, with China’s coreness being far greater than other countries. China thus functions as the absolute core of the entire BRI trade network. Except for Singapore, the other three countries are large countries with vast domestic markets that support the prosperous development of import and export trade. Singapore is a particular case because it is small but occupies an important core position in the trade network, mainly because of its port and policy advantages. Its re-export trade and unique location have thus allowed it to become an important node in the trade network. The countries in the semi-peripheral circle include Indonesia, the Czech Republic, Saudi Arabia, Romania, Vietnam, Ukraine, Lithuania, Croatia, Pakistan, Serbia, Egypt, and Malaysia. These 12 countries are mainly located in Southeast Asia and Central and Eastern Europe and have some trade influence in the local region but limited overall trade strength. The remaining BRI countries, 44 in total, are situated in the peripheral circle of the trade network due to various factors, such as domestic markets, industrial structure, economic strength, national volume, and geopolitical environment.

### 3.4 Backbone structure of the BRI trade network

**Fig 5:**
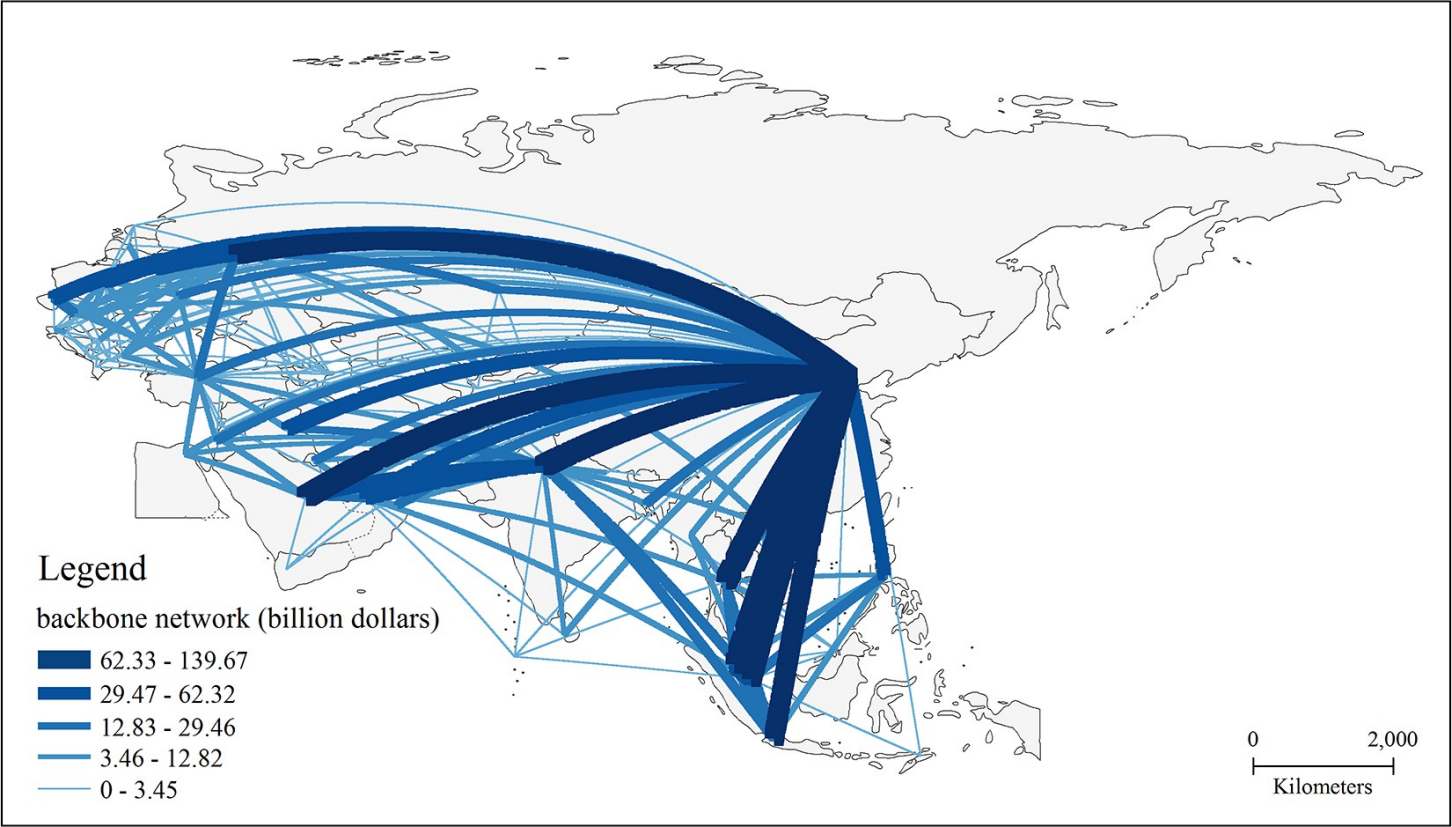
Backbone structure of the BRI trade network. Note: The basemap used to construct the map was obtained from the following source: China Map Publishing House Co., LTD. They have been republished under a CC BY license, with permission from the China Map Publishing House Co., original copyright [2021].

From the connectivity perspective, the disparity filter algorithm was used to eliminate the interference of an excessive number of edges in the original network, retain all the scale attributes and hierarchical structure, and further figure out the backbone framework of dominant connections in weighted networks. The backbone structure is the core structure of the whole network and supports the operation of the network. The backbone structure of the BRI trade network is shown in Fig 5. On the whole, the BRI trade network represents prominent hierarchical characteristics, showing a backbone network pattern with China as the absolute core, radiating outward and connecting the entire region, while India, Russia, and Turkey have also formed their backbone networks in local regions.

The backbone structure includes 221 trade relations, with apparent heterogeneity in spatial distribution, forming a hierarchical structure with a few nodes as the core. In general, the backbone structure of the BRI trade network has the following characteristics: 1) Trade links with China form the backbone structure of the trade network. More than 60 trade links have been established with China in the backbone structure, accounting for nearly one-third of the number of the entire trade links in the backbone network. Trade flows between China and Vietnam, China and Russia, China and Malaysia, China and Thailand, and China and India ranked among the top five in the backbone network. Among these dominant links, the trade links with more than 100 billion dollars are China-Vietnam, China-Russia, and China-Malaysia. Nine of the top 10 trade links in the backbone structure regarding trade flows are related to China. It shows that the trade flows between the BRI countries with China support the entire network structure. 2) Trade links related to energy trade and re-export trade are a vital part of the backbone structure of the trade network. In addition to China’s large-scale trade flows with the related countries, energy trade and re-export trade are the second tiers of trade links in the backbone structure of the BRI trade network. Among the top 20 trade links, 13 are connected to China, and the remaining seven are related to re-export trade and energy trade. Specifically, the trade links of Malaysia–Singapore, and Indonesia—Singapore are closely related to the re-export trade function of Singapore, while the trade links between UAE—India, India—Saudi Arabia, India—Iraq, and Russia—Turkey are all energy trade-oriented. 3) Russia, India, Turkey, UAE, Thailand, and Singapore are crucial nodes of the backbone structure. In the backbone structure, these countries are the key nodes after China, with more than 10 outward trade links, while all of these countries are among the top countries in the BRI region in terms of trade value. Russia, India, and Turkey are the most important regional trade centres, with 29, 27, and 22 trade links, respectively. Russia has strong trade links with China and Central and Eastern Europe, India has strong trade links with South Asia, Southeast Asia and the Middle East, while Turkey has become an important hub connecting Central and Eastern Europe, the Middle East and Central Asia. 4) Trade links between Central Eastern Europe countries are an important part of the BRI backbone network. In terms of the hierarchical structure, the strongest trade flows are concentrated among the countries, with China, India and Russia as the core. However, in the second hierarchical structure, the distribution of the backbone network is differentiated, especially in Central and Eastern Europe, where strong ties have been formed between countries within the region, and the trade blocs are clustered in geographical space. This also shows that the trade among Central and Eastern Europe countries in the BRI trade network has evident cohesion.

## 4. Discussion

Economic globalization and technological advances have transformed the world into a networked space, and global trade networks are considered defining features of contemporary globalization [34]. The connectivity of a node in a network determines the power and position of the node in the network. Therefore, assessing the trade network connectivity is essential to understanding the structure of trade networks, optimizing trade development patterns, and improving uneven development. However, in research scales, previous studies on trade networks have been dominated by macro- or micro-scale perspectives. The macro perspective of trade networks focuses on examining the whole connectivity characteristics of the network and reveals the overall topology and disequilibrium structure of trade networks. On the other hand, trade network studies from a micro perspective tend to explore the ranking and distribution of different nodes in the global or local trade network, portray the importance and influence of nodes in the network, and examine the trade network structure from the bottom up [35]. Although some studies have investigated trade networks from the approach of community detection or core-periphery structure [3,6], they often adopt a single research lens and fail to integrate multiple mesoscale structures to conduct an in-depth and systematic assessment of trade network connectivity.

As an emerging topic in network science, the mesoscale structure provides an analytical perspective on the inherent structural features embedded in networks, which is crucial for understanding and appreciating the network structural connectivity, and bridges the gaps in current network science [36,37]. Meanwhile, with the development and advancement of network analysis methods, complex network is becoming an essential perspective for studying trade networks, providing a more scientific and accurate analytical approach for exploring the international trading system. However, considering the global trade network’s complexity, hierarchy and non-equilibrium, it is still not a simple task to combine the structural properties of networks, select practical analytical algorithms and construct an analytical framework with suitability and stability to reveal trade network connectivity in depth.

In this study, we attempt to integrate multiple mesoscale structure-related algorithms, including community structure, core-periphery structure, and backbone structure, to construct an analytical framework for measuring trade network connectivity and take the trade network along the Belt and Road as an example. Compared with the previous studies, this study focuses more on the potential mesoscale structures embedded in the network from a connectivity perspective. The complementary approach integrates multiple mesoscale structure algorithms and helps understand the structural characteristics, topological relationships, unbalanced structure, and global and local relationships in real-world networks. Therefore, in the methodology, the analytical framework constructed in this paper for examining the connectivity of network structures from a mesoscale perspective has great potential to be widely applied to other disciplines and fields.

## 5. Conclusions

This paper attempts to integrate the community detection, core-periphery profile, and backbone structure algorithms and construct an analytical framework for assessing structural connectivity from a mesoscale perspective. Based on the analytical framework, we explore the structural connectivity of the BRI trade network, which is vital for optimizing the trade network structure and improving the sustainable development of trade cooperation. This study demonstrates the following findings:

1. In terms of trade volume, The trade network along the “Belt and Road” has formed a trade pattern of “one superpower, many great powers”, with three major trade clusters in Southeast Asia, the Middle East, and Northern Central and Eastern Europe in terms of geographical space. China is the super core of the trade network, and the most considerable trade connections are centred in China. In terms of trade flows, trade links between large economies in the BRI trade network are powerful, and the relations with the largest trade flows are centred on China. Extensive trade connectivities between the nodes of the BRI trade network prevail, even if the trade volumes between some countries are small.
2. In the context of the deep integration of globalization and regionalization, five distinctive trade groups have formed in the BRI trade network: China-Southeast Asia Cluster, Russia-Central Asia-Eastern Europe Cluster, South Asia—Eastern and Southern Middle East Cluster, Turkey-Northern and Central Middle East Cluster, Central and Southeastern Europe Cluster. Nevertheless, the structure of trade groups shows significant geographical proximity, indicating that geographical distance still plays a vital role in the international trade system at the regional scale.
3. The concentration coefficient of the BRI trade network indicates that the BRI trade network demonstrates a significant core-periphery structure, with apparent trade clustering among the core countries within the trade network. The BRI countries can be divided into core, semi-peripheral, and peripheral structures. The core structure of nine countries led by China constitutes China, India, Russia, Singapore, Poland, Thailand, Turkey, the United Arab Emirates, and Hungary. The semi-peripheral structure includes Indonesia, the Czech Republic, Saudi Arabia, Romania, Vietnam, Ukraine, Lithuania, Croatia, Pakistan, Serbia, Egypt, and Malaysia, for 12 countries. Due to the various factors such as domestic markets, industrial structure, economic strength, national volume, and geopolitical environment, the number of countries in the peripheral structure is large, reaching forty-four.
4. The backbone structure includes 221 trade relations, with a clear hierarchy, closely connected, and outstanding core connective features. The trade links with China constitute the backbone of the whole trade network. In addition, the trade links related to energy trade and re-export trade are also crucial components of the BRI backbone structure. Russia, India, UAE, Thailand, and Singapore are also important nodes of the backbone structure.

The BRI aims to share the fruits of human development among a wider range of countries and a larger population. However, as for the structural connectivity of the BRI trade network, the issue of uneven regional development is still prominent. Although the BRI economic and trade cooperation is full of opportunities and potential, BRI cooperation still faces more challenges and problems. In the future, the construction of the BRI should focus more on the following strategies: 1) The BRI countries should firmly promote the direction and intensity of economic and trade cooperation so that the benefits of trade development can spread to more countries and people along the BRI route. 2) The BRI should further improve trade connectivities between countries and regions, expand trade volume, and optimize trade structure. 3) The BRI should strengthen infrastructure connectivity among countries along the BRI route and promote trade cooperation facilitation and development. 4) The BRI should promote establishing a higher level and broader range of preferential free trade policy systems at the right time to further lower trade barriers, reduce trade disputes, and stimulate cooperative development and mutual benefits brought by international trade.

## Supporting information

S1 File (CSV)

S2 File (CSV)

S3 File (CSV)

S4 File (CSV)

S5 File (CSV)
